# Inferring epidemiological links from deep sequencing data: a statistical learning approach for human, animal and plant diseases

**DOI:** 10.1098/rstb.2018.0258

**Published:** 2019-05-06

**Authors:** M. Alamil, J. Hughes, K. Berthier, C. Desbiez, G. Thébaud, S. Soubeyrand

**Affiliations:** 1 BioSP, INRA, 84914 Avignon, France; 2 MRC-University of Glasgow Centre for Virus Research, Glasgow G61 1QH, UK; 3 Pathologie Végétale, INRA, 84140 Montfavet, France; 4 BGPI, INRA, Univ. Montpellier, SupAgro, Cirad, 34398 Montpellier, France

**Keywords:** contact information, infectious disease, pathogen spread, training data, transmission trees, within-host pathogen diversity

## Abstract

Pathogen sequence data have been exploited to infer who infected whom, by using empirical and model-based approaches. Most of these approaches exploit one pathogen sequence per infected host (e.g. individual, household, field). However, modern sequencing techniques can reveal the polymorphic nature of within-host populations of pathogens. Thus, these techniques provide a subsample of the pathogen variants that were present in the host at the sampling time. Such data are expected to give more insight on epidemiological links than a single sequence per host. In general, a mechanistic viewpoint to transmission and micro-evolution has been followed to infer epidemiological links from these data. Here, we investigate an alternative approach grounded on statistical learning. The idea consists of learning the structure of epidemiological links with a pseudo-evolutionary model applied to training data obtained from contact tracing, for example, and using this initial stage to infer links for the whole dataset. Such an approach has the potential to be particularly valuable in the case of a risk of erroneous mechanistic assumptions, it is sufficiently parsimonious to allow the handling of big datasets in the future, and it is versatile enough to be applied to very different contexts from animal, human and plant epidemiology.

This article is part of the theme issue ‘Modelling infectious disease outbreaks in humans, animals and plants: approaches and important themes’. This issue is linked with the subsequent theme issue ‘Modelling infectious disease outbreaks in humans, animals and plants: epidemic forecasting and control’.

## 1. Introduction

In order to most effectively predict and control the spread of infectious diseases, we need to better understand how pathogens spread within and between host populations and assess the role of the environment in the transmissions. The question *how do pathogens spread?* can be understood in many ways. Here, we consider the case where we observe numerous host units infected by an endemic or epidemic infectious disease, and the question of *how do pathogens spread?* translates into *who infected whom?* or *who is closely related to whom?* in the disease transmission dynamics. Host units typically designate individuals but can also designate groups such as households, premises and agricultural fields.

For fast-evolving pathogens, numerous approaches exploiting pathogen sequence data have been developed with the aim of inferring who infected whom or who is closely related to whom. These approaches are grounded on a wide variety of principles, from those based on statistical metrics to those based on a mechanistic modelling of pathogen transmission and micro-evolution. For instance, transmission links can be inferred by identifying specific variants shared by different hosts or minimizing differences in single nucleotide polymorphisms (SNP) [1–3], by combining minimal genetic distances between intra-host viral populations and properties of social networks relevant to pathogen spread [4], by applying methods based on phylogeny, phylogeography and some forms of birth–death processes [5–14], or by using methods based on joint models of epidemiological dynamics and evolutionary processes [15–21]. Initially, model-based approaches mostly exploited a single pathogen sequence per host. Nevertheless, the progress of sequencing techniques revealing the within-host genetic polymorphism of pathogens fostered the development of model-based approaches accounting for the generation of within-host diversity and/or leveraging the information provided by sets of sequences sampled from hosts [4–7,9,14,20].

Approaches based on a mechanistic vision of transmission and micro-evolutionary processes are the most obvious direction to follow for inferring epidemiological links between host units. Indeed, mechanistic assumptions underlying these approaches act as relevant constraints, which are expected to guide the inference. However, statistical learning techniques [22] adapted to the inference of epidemiological links should also be developed, in particular (i) when mechanistic assumptions could be inadequate and, therefore, misleading, (ii) when sequence data do not accurately reflect the within-host pathogen population because of sequencing bias or errors and (iii) when a fast method is required to tackle big datasets in terms of number of hosts, sequencing depth and sequence length.

Here, we propose a statistical learning approach for estimating epidemiological links from deep sequencing data (called SLAFEEL), which is based on a parsimonious semi-parametric pseudo-evolutionary model. This model is designed as a regression function where the response variable is the set of sequences **S** observed from a recipient host unit and the explanatory variable is the set of sequences **S**_0_ observed from a putative source. The coefficients of the regression are weights measuring how much each sequence in **S**_0_ contributes to explaining each sequence in **S**. These weights account for the gain and loss of virus variants during within-host evolution and their loss during between-host transmission. The model is semi-parametric because it depends both on parameters and on a kernel smoother (a tool from non-parametric statistics), which accounts for unsampled sequences in the source of infection, the evolution of new viral variants and potential sequencing errors. The model is pseudo-evolutionary because, even if it does not explicitly model evolutionary processes, it contains terms that macroscopically reflect these processes. From this model, we built a penalized pseudo-likelihood, which is used for selecting who infected whom (or who is closely related to whom). Two hypotheses (H1 and H2) were considered for the penalization. H1: The penalization assesses whether the contributions of sequences in **S**_0_ to explain sequences in **S** are homogeneous (two penalization shapes were introduced in this case: H1-normal and H1-*χ*^2^). H2: The penalization assesses whether the distance between sequences in **S** and their contributing sequences in **S**_0_ is consistent with some known features, e.g. with an expected value for this distance (one penalization shape was introduced in this case: H2-normal). In both cases, a penalization parameter measures the strength of the penalization, and this parameter is calibrated with training data. In the epidemiological contexts tackled in this study, training data consist of contact tracing (who has been in contact with whom) or geographical distances between host units (that can be viewed as a contact proxy). Contact information has to be available only for a subset of hosts, hereafter called *training hosts*. Finally, for each putative donor–recipient pair, our method provides a link intensity measuring whether the set **S**_0_ collected from the putative donor likely explains the set **S** collected from the recipient. In addition, the link intensities can enable an assessment of the uncertainty of the reconstruction of donor–recipient links.

In what follows, we pave the way for this statistical learning approach aiming at inferring transmissions of infectious diseases (caused by fast-evolving pathogens) from deep sequencing data, and we apply it to three real cases in animal, human and plant epidemiology. The animal case study concerns swine influenza virus (SIV) and here serves as a test study since the transmission chain is partly known. The human case study, dealing with Ebola, is a particularly challenging situation since little diversity is observed in the pathogen population and limited contact tracing information is available. The plant case study concerns a potyvirus of wild salsify transmitted by aphids where the host unit is the meadow. In this latter application, we are more interested in estimating who is closely related to whom than who infected whom. The generic nature of SLAFEEL allows dealing with diverse epidemiological situations and sequencing procedures, as illustrated by the three case studies and in §3 of this article.

## 2. Results

### (a) Tracing experimental swine influenza outbreaks

The first dataset was generated from an experimentally controlled transmission chain of SIV in pigs with different immunological histories (naive and vaccinated; [2]). For each chain, pairs of pigs were successively settled in an experimental enclosure, with a temporal overlap between the arrival of the new pair and the departure of the preceding pair to allow the virus to be transmitted. Thus, the infection pathways are partly known and will be used to assess the efficiency of SLAFEEL. For each pig, the virus population was sampled on a daily basis, and multiple clones of the hemagglutinin gene were sequenced using a capillary approach (Sanger sequencing). The naive chain consisted of five pairs of pigs from which 21 samples of the viral populations were collected with multiple time points for eight pigs. The vaccinated chain consisted of seven groups of pigs from which 29 samples of the viral populations were collected with multiple time points for seven pigs. Further details about the SIV dataset are provided in electronic supplementary material, table S1.

Transmission chains were inferred for the two experimental outbreaks with SLAFEEL. The penalization was calibrated for each outbreak with contact information from two *training* hosts, which were either the two pigs of the last group of the outbreak or a pig from the third group and a pig from the fourth group. The training hosts and the hosts with which they have been in contact, including the host in the same group, are detailed in electronic supplementary material, table S2. For this application, we chose the H1-normal penalization (see §4b) that led to higher consistency between contact information and inferred transmissions. For each host, the *response* set of sequences was the first sample collected from this host, and the potential *explanatory* sets of sequences were every sample collected earlier or at the same time from all the other hosts.

**Figure 1.**
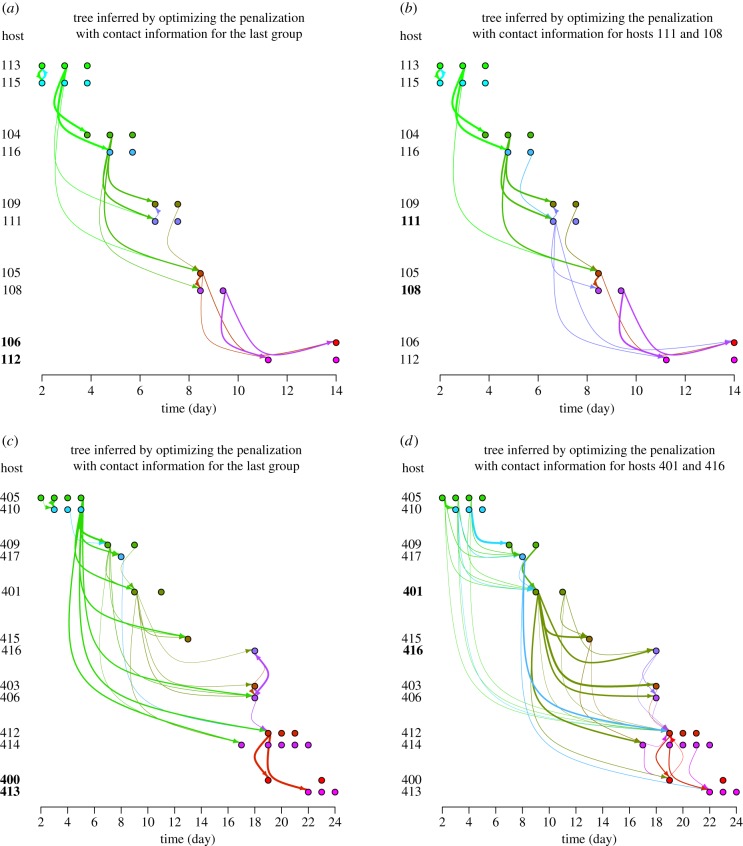
Transmissions inferred in the naive and vaccinated chains with two different pairs of training hosts for calibrating the penalization. Panel (*a*) corresponds to the naive chain using pair 106–112 as training hosts (i.e. the last group of the chain); (*b*) naive chain, pair 111–108; (*c*) vaccinated chain, pair 400–413 (i.e. the last group of the chain); (*d*) vaccinated chain, pair 401–416. Training hosts are written in bold. The thickness of each arrow is proportional to the intensity of the corresponding inferred link. (Online version in colour.)

Figure 1 shows transmissions inferred with SLAFEEL for the naive and vaccinated chains. For the naive chain, we observe rather consistent estimations with the two pairs of training hosts, even if we observe variation in secondary links with low intensities displayed with thin arrows (the link intensity measures the likelihood of the link; see §4c). By contrast, for the vaccinated chain, the training hosts have an impact on the inference. Indeed, the use of training hosts in the last group leads to the identification of many indirect links as transmissions, whereas the use of training hosts in the middle of the chain reduces this shortcoming (even if the sources for hosts 403, 406, 412 and 414 remain inadequately inferred). Electronic supplementary material, figure S1 shows how this uncertainty is also reduced by adding a third training host to the last group. Using more contact information allows a finer calibration of the penalization (electronic supplementary material, figure S2) and, consequently, a more accurate resolution of transmissions. Moreover, the advantage of introducing a penalization is clearly illustrated by electronic supplementary material, figure S3, which displays transmissions estimated without penalization: for the naive chain, host 113 is erroneously identified as the source of infection of numerous hosts.

### (b) Inferring Ebola epidemiological links despite low pathogen diversity

In this section, we analyse the dataset generated during the 2014 Ebola virus disease (EVD) outbreak in Sierra Leone [23]. We were able to include in our analysis 58 confirmed EVD patients, from which within-host populations of the virus were collected and sequenced. This number of patients represents nearly 50% of the EVD patients diagnosed in Sierra Leone from late May to mid-June. Viral populations were sequenced using the Nextera library construction method and Illumina sequencing and the haplotypes were estimated in a sliding window of 1000 bases every 500 bases using Predict-Haplo [24].

More details about the Ebola dataset are provided in electronic supplementary material, table S1. Here, we simply highlight the rather low pathogen diversity that was observed: on average, 16.1 haplotypes per fragment of 1000 bases were identified for the 58 patients included in the analysis (s.d. = 8.0), and 1.37 haplotypes per fragment of 1000 bases per patient (s.d. = 0.64).

Epidemiological links between patients were inferred by calibrating the penalization with contact tracing published in [25]. We were able to use five donor–recipient *training* pairs identified with contact tracing (see electronic supplementary material, table S2), four of them having the same putative donor. For this application, we chose the H2-normal penalization (see §4b), which led to higher consistency between contact information and inferred transmissions in a situation where observed pathogen populations show relatively low levels of diversity. Several samples were available for some of the patients collected at different time points [23]. These samples were merged in our analysis to increase the within-host sequence diversity. In addition, we applied the statistical learning approach separately for 31 partly overlapping fragments of 1000 nucleotides, and we aggregated the results for reconstructing the epidemiological links. For each host, potential sources were inferred among patients observed earlier than or at the same time as the target host (point discussed in §3).

**Figure 2.**
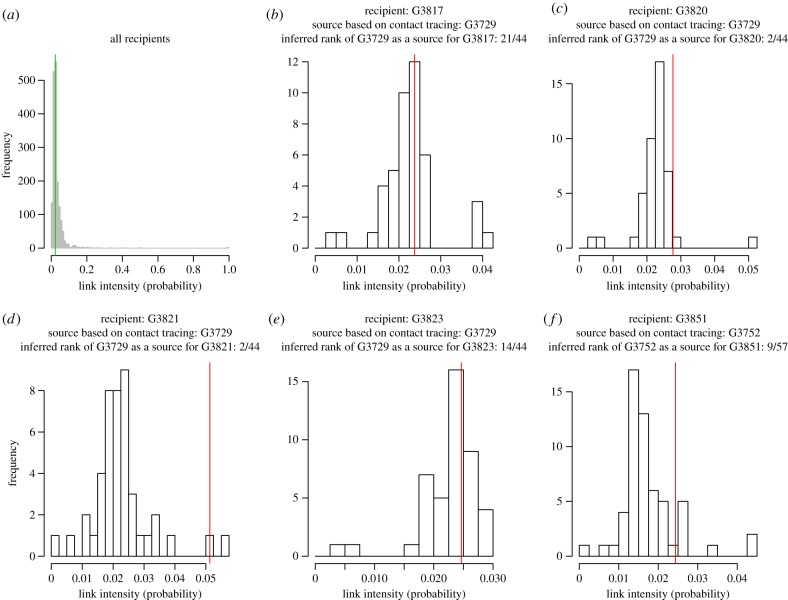
Estimated intensities of links for all recipients (*a*; vertical line: median intensity) and for each recipient in the training set of hosts (*b*–*f*; vertical lines: intensity for the source identified with contact tracing). This figure was obtained from the combined analysis of 31 sequence fragments and with cross-validation. Analogous figures obtained without cross-validation and with half of the fragments are given in electronic supplementary material, figures S6–S8. The second half of fragments led to approximately the same results for training hosts. Note in addition that using only one fragment for inferring transmissions led to particularly stochastic outputs. (Online version in colour.)

**Figure 3.**
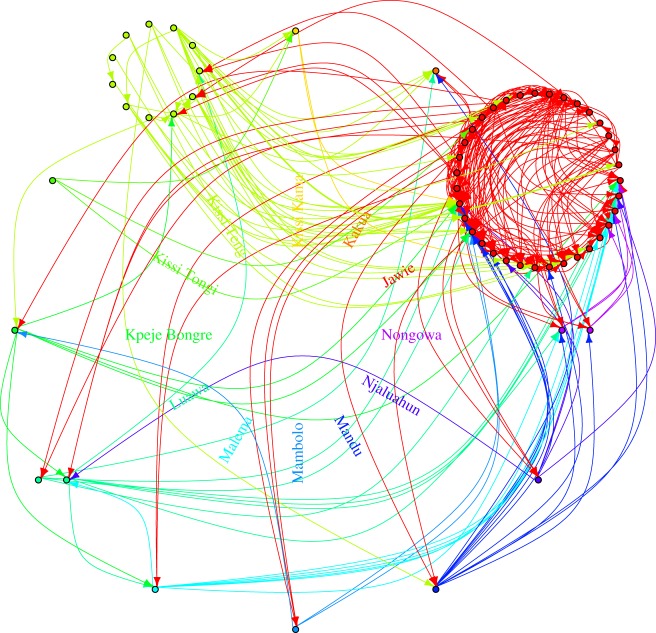
Most likely epidemiological links cumulating to 20% probability for each recipient (i.e. for each recipient, potential donors were ranked with respect to link intensity, and the subset of donors with higher ranks for whom the sum of link intensities reached 0.2 were displayed on the graph). (Online version in colour.)

Because of the reduced pathogen diversity, the inferred intensities of epidemiological links are generally quite low (figure 2*a*) and multiple sources for any host are plausible (except those at the earlier time points of sampling for which only a few potential sources are allowed). Thus, source identification is quite uncertain. Figure 2*b*–*f* shows the distributions of the link intensities with plausible sources for the five recipients in the training data, and give the ranks of their sources identified with contact tracing. The intensities and ranks were inferred with a leave-one-out cross-validation approach (i.e. the host of interest in each panel is removed from the training data when one infers its source and the rank of its donor based on contact-tracing). The donors identified with contact tracing are well ranked for patients G3820, G3821, G3823 and G3851, but not for G3817. The pathogen population collected from the latter patient is actually quite different from the population observed in its putative donor G3729 (see electronic supplementary material, table S3, and the Ebola phylogeny built from the consensus sequences [26]). Thus, the epidemiological link between G3817 and G3729 could be revisited by focusing on patients who are more closely connected to G3817 than G3729 (see electronic supplementary material, tables S4–S8). Figure 3 displays the most likely epidemiological links cumulating to 20% of probability for each recipient (see figure caption). Patients are clustered based on their chiefdoms, whose locations are provided in electronic supplementary material, figure S4. The Jawie chiefdom seems to be an interface between Kissi Teng and Kissi Tongi chiefdoms on the one hand and most of the other chiefdoms on the other hand. Based on temporal data (electronic supplementary material, figure S5), the Kissi Teng and Kissi Tongi chiefdoms include mostly early cases and, therefore, individuals in Jawie chiefdom may have played the role of a relay in the outbreak.

### (c) Assessing epidemiological links at the metapopulation scale

This dataset was generated from a wild plant species (*Tragopogon pratensis*, hereafter called wild salsify), which is a reservoir for a potyvirus closely related to the endive necrotic mosaic virus (ENMV; [27]). Within-host virus variants were sequenced from 189 infected host plants sampled in 2014 in a 40×10 km region of south-eastern France. High-throughput sequencing was applied on viral PCR amplicons (final length: 438 bp of the capsid gene) using the Illumina technology [28]. Sequence data were merged at the scale of the patch (i.e. meadows, agricultural fields or urbanized areas) with the aim of assessing epidemiological links between a subset of the metapopulation formed by the potyvirus (the 189 sampled plants were distributed in 27 patches). Further details about this dataset are provided in electronic supplementary material, table S1.

Epidemiological links between sampled patches were inferred by calibrating the penalization with information on inter-patch distances, assuming that, on average, geographically close host patches are infected by similar viral variants (isolation-by-distance process). Here, the H1-*χ*^2^ penalization (see §4b) was chosen because it led to a lower average distance between connected patches (see criterion (4.7), §4c).

**Figure 4.**
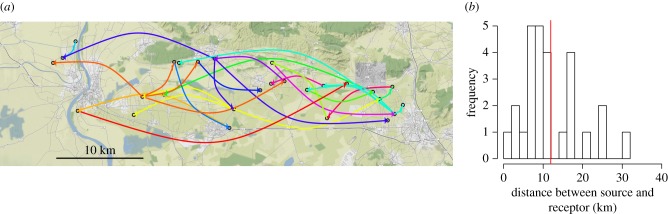
Links inferred between salsify patches based on sampled sets of potyvirus sequences (*a*; links from the same source have the same colour) and distribution of link distances (*b*; the vertical red line gives the mean distance). (Online version in colour.)

Figure 4 shows the inferred links between sampled patches. Here, all the optimal values for the penalization parameter (shown in electronic supplementary material, figure S9) led to the same set of links and, therefore, no secondary arrows are displayed (electronic supplementary material, figure S10 shows links inferred without penalization). Even if most links are relatively short compared to the mean distance between sampled patches (see electronic supplementary material, figure S11), there is a non-negligible proportion of long links that could be the signature of the long-distance dispersal ability of the aphid to transmit the virus. Additionally, common environmental conditions and host demography and genetics at the scale of the study area may partly explain the inferred long-distance links. Indeed, environmental conditions constrain host local abundance and, therefore, genetic drift impacts on the levels of diversity and differentiation within and between local pathogen populations. Spatial variation in host genetics may also shape the spatial structure of pathogen populations by selecting different variants regardless of the distance between host patches [29,30].

### (d) Benchmarking SLAFEEL

We first compared SLAFEEL and BadTrIP [5] for influenza data to assess the ability of both methods to identify infection pathways that are partly known. Electronic supplementary material, figure S12, gives details about the application of BadTrIP and shows inferred transmission trees. Whatever training hosts were used, SLAFEEL generally performed better than BadTrIP with respect to the proportion of correct source identifications (that focuses on the most likely inferred source) and the average Jeffreys discrepancy (that compares the probabilities for any recipient host to be linked with any putative source) as presented in electronic supplementary material, table S10.

Second, we compared the transmissions inferred with SLAFEEL from the Ebola data and those obtained in [5] with BadTrIP. Here, we assessed the consistency of both estimations (since potential infection pathways are not known, unlike in the influenza case study). The most likely sources are the same for 8% of recipient hosts (electronic supplementary material, table S10) and the most likely sources inferred with SLAFEEL are among the 10 most likely sources identified with BadTrIP for almost 50% of recipients (electronic supplementary material, figure S13). These rather low percentages may be explained by the low pathogen diversity in this study, leading to generally quite low inferred link intensities with SLAFEEL and, to a lesser extent, with BadTrIP (see electronic supplementary material, figure B in [5]). They may also be explained by the assumptions made and the constraints imposed in [5], where information from sampling dates, nucleotide frequencies and sequencing coverage was used, and where the introduction date (removal date) of each host was specified as its sampling date minus (plus) 21 days, thus allowing each host to be infected at most 21 days before being sampled, and to infect others at most 21 days after being sampled.

Finally, we simulated 1000 datasets with the SEEDY package (simulation of evolutionary and epidemiological dynamics; [20]) by using parameter values chosen by Worby and Read to generate their 4th figure (mean epidemic size: 26.6 infected hosts (s.d. = 2.3); 10 virus genomes sampled per host). The SEEDY package allows not only the generation of datasets, but also a very fast inference of transmissions given infection times, the mutation rate, the equilibrium viral population size within host and the transmission bottleneck size, which are generally not known in practice. Thus, we used SEEDY-based inferences of transmissions as a benchmark, and assessed how SLAFEEL compares with SEEDY in identifying the true source for each recipient of each of the 1000 simulated outbreaks. For the application of SLAFEEL to each simulated outbreak, we randomly drew four training hosts whose sources were supposed to be known, and we chose the H1-normal penalization. On average, the most likely inferred source was correct for 39% [20–61%] of recipients with SEEDY and 36% [17–60%] with SLAFEEL (electronic supplementary material, figure S14). Therefore, in this simulation setting, SLAFEEL performs almost as well as SEEDY.

## 3. Discussion

We introduced an exploratory approach, called SLAFEEL, for quantitatively investigating epidemiological links between host units from deep sequencing data. This versatile approach, grounded on statistical learning, is adaptable to diverse contexts and data. Here, we applied it to analyse virus dynamics in humans, animals and plants at different spatial scales (e.g. individuals and fields) using data obtained with different sequencing techniques and showing different levels of pathogen diversity. The relatively broad applicability of SLAFEEL implies that, in some contexts, links have to be interpreted in a conservative way: typically, in the salsify potyvirus application, we did not infer *who infected whom* but *who is closely related to whom*. Using the pseudo-evolutionary model and the associated inference approach for estimating epidemiological links should be particularly valuable in non-standard situations where classical mechanistic assumptions may be erroneous and when sequencing and variant calling issues may be misleading. The key property underlying our procedure is the combination of a learning stage and a penalization that can be used to constrain what is a link. This is expected to help in appropriately dealing with sequencing errors because such errors should be accounted for non-training hosts as they are for training hosts. Nevertheless, as discussed below, the impact of sequencing errors on inference accuracy should be formally assessed in simulation studies.

The training stage can use classical information such as contact tracing data [25], but also contact proxies such as geographical distances between host units, connectivities via air masses for airborne pathogens [31] and social connections [4,32]. To get a contact proxy, one could also infer some transmissions with a (generally more time-consuming) mechanistic approach from a subset of observed cases and use the estimated transmissions as training data in our approach applied to the whole dataset. Thus, the mechanistic approach and SLAFEEL would be complementary. Whatever the way that contact information (or proxies) are gathered, it can be conjectured that the closer the relationship between contact information and epidemiological links, the more informative the training stage. Moreover, the possibility of using very diverse types of contact information in the learning stage of SLAFEEL reinforces its broad relevance to human, animal and plant diseases.

When geographical proximity is used for calibrating the penalization (like in the potyvirus application), short-distance links may be favoured, and the inferred distribution of distances between linked host units hence has to be interpreted with caution. However, in our procedure, geographical proximity is only used after a genetic-based selection of possible configurations: basically, the penalized pseudo-likelihood function (only based on virus sequence data) allows us to eliminate genetically unlikely configurations; then, in the learning stage, spatial information is used to select the most likely configurations within the set of genetically likely configurations, building on the following grounds: among two equally genetically likely configurations, the one showing links at shorter distances is more likely (because of the very classical assumption that ‘dispersal is more probable at short distance than at long distance’). Thus, inferring only short-distance links can be interpreted as: ‘short distance dispersal is sufficient to explain the genetic spatial pattern of the pathogen’. By contrast, inferring both (i) a mixture of short- and long-distance links and (ii) unlinked nearby host units (like in the potyvirus application) suggests that isolation by distance does not hold at the study scale, and that the assumption ‘dispersal is more probable at short distance than at long distance’ is perturbed by other drivers (e.g. host genetics), which significantly impact the genetic spatial pattern of the pathogen. Finally, while our analysis in the potyvirus application leads to interpretable results, cross-validation or data-splitting (into training and prediction data) could be applied in further studies to strengthen the analysis conclusions when geographical proximity is used as contact information.

The main objective of this article was to present how statistical learning can be applied for inferring transmissions (or epidemiological links from a conservative perspective) and to examine if such an approach has the potential to be efficient. Results obtained for swine influenza (where the transmission pathways are partly known) and for outbreaks simulated with SEEDY [20] are encouraging. However, further research is required to make the method robust and able to pass a battery of simulation tests such as the one designed for assessing the performance of BadTrIP [5]. The following questions should be specifically investigated using simulations. How does the efficiency and speed of the method scale up with big data? How does the method perform at various sequencing depths (considering a single haplotype for each host as a special case)? How does the method perform in the presence of contamination and sequencing errors (PHYLOSCANNER [14] explicitly handles such issues)? What is the sensitivity of the method to the haplotype reconstruction tool (e.g. comparing Predict-Haplo that we used for the Ebola data with SAVAGE [33] and MLEHaplo [34])? How is SLAFEEL accuracy improved with increasing training information? How can we exploit negative training information (i.e. infected hosts that are known to not have been in contact with certain infected hosts)? How does the method perform in the presence of severe bottlenecks during transmissions, in comparison with approaches exploiting phylogenetic signals that are particularly adapted to such situations [9]?

Before testing SLAFEEL in the latter range of simulation settings, further research should especially focus on the penalization function. Here, we introduced three shapes corresponding to different hypotheses (see §4b), but the penalization could be tuned by considering other hypotheses, which could help circumvent the current limitations of our approach. For instance, the penalization could be improved to take into account (i) the timing, thus constraining the set of likely sources for each host based on observation times and possibly additional temporal information like data on infectious periods [17], (ii) fixed sub-clonal haplotypes (including haplotypes with stop codons) by forcing the selection algorithm to pair host units sharing such haplotypes [1,35] and (iii) sample sizes to avoid biases induced by different levels of observed diversity. Specific penalizations could also be designed to better infer the direction of epidemiological links when temporal data do not discriminate sufficiently. For example, the signature of the link direction could be identified in the genetic training data and incorporated into the penalization function. Other limitations are more difficult to tackle, e.g. de novo mutations at the same site (homoplasy), recombinations, insufficient sequencing depth and lack of sequence diversity, which can lead to uncertainty in the inferences. However, the advantage of our statistical learning approach is that the uncertainty can be objectively assessed on training data. The uncertainty (and potential bias) can even be assessed using cross-validation to prevent over-fitting. The assessment of uncertainty and bias in the inference of links is also an objective way to select the penalization shape. However, we must warn that, if training data are not representative of the whole population, learning model parameters from training data may induce errors in the selection of the penalization and, ultimately, in the reconstruction of epidemiological links (such misleading training data would be analogous to misleading assumptions in mechanistic approaches).

Another important perspective is the implementation of an efficient computer code. The R code that we developed (available at https://doi.org/10.5281/zenodo.1410438) allowed us to test different model specifications, to exploit genetic data from multiple sequence fragments and to perform cross-validation in a limited time-span (e.g. a SLAFEEL run for the swine influenza case study or for a sequence fragment in the Ebola case study took approximately 10–20 minutes with a laptop computer, whereas BadTrIP takes several days; see caption of electronic supplementary material, figure S12 and [5]). However, implementing further improvements in the code should allow us (i) to include multiple infections in transmission scenarios where an *explanatory* set of sequences would consist of a weighted mixture of several samples collected from several putative sources, (ii) to select a penalization shape among a large library of functions, and (iii) to tackle big data (e.g. large numbers of cases and sequence fragments). Concerning point (iii), our approach based on a simplified representation of dependencies between observations via a statistical regression model is a commonly used approach to handle big data [36].

## 4. Methods

To infer transmissions of a virus (or, more generally, epidemiological links) within a host population, we built a pseudo-evolutionary model that concisely describes transitions between sets of sequences sampled from different host units, and used this model to select probable source–recipient pairs. In what follows, we provide the outline of our method in one of its simplest forms (see also electronic supplementary material, figure S15), then we technically describe it in its general form by presenting first the model and second the inference.

### (a) Outline of the SLAFEEL approach

Let us consider one of the possible source–recipient pairs. For each virus sequence collected from the recipient, we compute the genetic distance (namely, the number of different nucleotides) to each sequence collected from the source, and we identify the nearest sequence(s). By applying this procedure to all sequences from the recipient, we can compute the contribution of each sequence from the source to *explain* the viral population observed from the recipient. This contribution relates to the number of times that this sequence from the source is identified as the nearest sequence (see the exact definition in §4b). Then, a parametric kernel function, derived from the Jukes–Cantor micro-evolutionary process and embedded in a pseudo-likelihood, is used to assess how much each sequence from the recipient is *explained* by its nearest sequence(s) from the source. Moreover, a parametric penalization function is used to assess how likely sequences from the source have been *uniformly* subsampled to generate sequences from the recipient (this is assessed based on the contributions calculated above). Thus, for each possible source–recipient pair, we compute a penalized pseudo-likelihood parameterized by the kernel parameter *μ* and the penalization parameter *θ*. The penalized pseudo-likelihood will be high for a putative source–recipient pair if (i) all sequences from the recipient have genetic neighbours in the source and (ii) sequences from the source equally contribute in expectation to the set of sequences collected from the recipient. Note that condition (ii) depends on the rationale underlying the form chosen for the penalization function (here, the penalization is grounded on a uniform subsampling hypothesis).

The balance between the pseudo-likelihood and the penalization is tuned in two steps. First, we estimate *μ*, for each source–recipient pair and each *θ* value in a set *Θ* of candidate values, by maximizing the penalized pseudo-likelihood with respect to *μ*; then, for each recipient and each *θ* value, the source leading to the maximum penalized pseudo-likelihood is identified as the most likely source given *θ*. Second, adopting a learning approach, we calibrate the penalization by selecting the *θ* values leading to the maximum proportion of *training hosts* for which the most likely sources conditional on *θ* are consistent with contact information. The link intensity between a given recipient and a possible source is measured by the proportion of selected *θ* values for which the source has been identified as the most likely source.

The dual form of the penalized pseudo-likelihood and the learning stage are essential to distinguish ‘A infected B’, ‘B infected A’ and ‘C infected B’ when only the former statement is true. Indeed, the pseudo-likelihood tends to impose that each sequence from the recipient must have a neighbour sequence in its source, which should exclude ‘C infected B’; the penalization tends to impose that the set of sequences from the recipient has been generated by a subsample of the set of sequences from the source (if the penalization has been built in this way), which should exclude ‘B infected A’; the learning stage is expected to determine the adequate relative weights of the pseudo-likelihood and the penalization for obtaining satisfactory inference of epidemiological links. The learning stage can even be exploited to design an adequate penalization form (one should prefer a penalization form leading to higher inference accuracy for training hosts).

### (b) Pseudo-evolutionary model for the evolution and transmission of populations of sequences

The method outlined above is grounded on a pseudo-evolutionary model, which concisely describes transitions between sets of sequences sampled from different host units. The general form of the pseudo-evolutionary model is given by the following penalized pseudo-likelihood for the transition from an explanatory set of *I* sequences $S_{1}^{\left(0\right)},\ldots ,S_{I}^{\left(0\right)}$ to a response set of *J* sequences *S*_1_, …, *S*_*J*_ (haplotype copies are explicitly incorporated in these sets of sequences):

4.1 $$f(S_{1},\ldots ,S_{J}|S_{1}^{\left(0\right)},\ldots ,S_{I}^{\left(0\right)})=P(W)\prod_{\;j=1}^{J}\left(\frac{\sum_{i=1}^{I}w_{ij}K(d(g(S_{\;j}),g(S_{i}^{\left(0\right)})))}{\sum_{i=1}^{I}w_{ij}}\right),$$

where each term in the product represents the pseudo-probability of obtaining the response sequence *S*_*j*_ given the explanatory sequences $S_{1}^{\left(0\right)},\ldots ,S_{I}^{\left(0\right)}$ and the values of *w*_1*j*_, …, *w*_*Ij*_; *g* is a transformation of sequences (e.g. aiming at reducing the dimension of the space of viral sequences); *K* is a kernel function and *d* is a pseudo-distance function introduced to account for unsampled sequences in the source of infection, the evolution of new viral variants and possible sequencing errors; *w*_*ij*_ are weights accounting for the loss of virus variants during within-host evolution and between-host transmission; *W* is the (*I* × *J*)-matrix of weights whose element (*i*, *j*) is *w*_*ij*_; and *P*(*W*) is a penalty for the weight matrix *W* potentially allowing the incorporation of knowledge on virus evolution and transmission (e.g. on the strength of the transmission bottleneck).

In this article, we focus on a simple semi-parametric version of (4.1) where (i) each sequence *S*_*j*_ is only explained by the closest sequence(s) $S_{i}^{\left(0\right)}$ in terms of the number of different nucleotides and (ii) the penalization measures the discrepancy from a null hypothesis to be specified. Thus, the pseudo-evolutionary model given by equation (4.1) reduces to:

4.2 $$\begin{array}{rl} f_{\mu ,\theta }\;(S_{1},\ldots ,S_{J} & |S_{1}^{\left(0\right)},\ldots ,S_{I}^{\left(0\right)})\\ & =P_{\theta }(W)\prod_{\;j=1}^{J}\left(\frac{\sum_{i=1}^{I}w_{ij}K_{\mu }\{d(S_{\;j},S_{i}^{\left(0\right)});\Delta_{ij}\}}{\sum_{i=1}^{I}w_{ij}}\right), \end{array}$$

where d(⋅,⋅) gives the number of different nucleotides between two sequences; *w*_*ij*_ = 1/*n*_*j*_ for indices *i* corresponding to sequences $S_{i}^{\left(0\right)}$ minimally distant from sequence *S*_*j*_, i.e. such that $d(S_{\;j},S_{i}^{\left(0\right)})=\min\{d(S_{\;j},S_{i^{\prime }}^{\left(0\right)})\;:\;i^{\prime }=1,\ldots ,I\}$, the number of such sequences being denoted *n*_*j*_, *w*_*ij*_ = 0 otherwise (therefore, $\sum_{i=1}^{I}w_{ij}=1$); Δ_*ij*_ is the duration separating the two sequences *S*_*j*_ and $S_{i}^{\left(0\right)}$; $K_{\mu }(\cdot ;\Delta )$ is the probability distribution function (p.d.f.) of the binomial law with size *L* (i.e. sequence length) and success probability 3(1 − exp (−4*μ*Δ))/4, corresponding to the Jukes–Cantor micro-evolutionary process over a duration Δ and with a substitution parameter *μ*; and $P_{\theta }(W)$ is a parametric penalization measuring the likelihood of the contributions of explanatory sequences $S_{1}^{\left(0\right)},\ldots ,S_{I}^{\left(0\right)}$ (measured by $\sum_{\;j=1}^{J}w_{ij}$, *i* = 1, …, *I*) to the response set of sequences *S*_1_, …, *S*_*J*_. If $\sum_{\;j=1}^{J}w_{ij}=0$, then sequence $S_{i}^{\left(0\right)}$ does not contribute to explaining the sequences collected from the recipient and, therefore, may be considered as lost during within-host evolution or between-host transmission.

We consider the three following shapes for $P_{\theta }$. The H1-normal shape measures the discrepancy between $\sum_{\;j=1}^{J}w_{ij}$ and its expected value *J*/*I* under the uniform (but not necessarily independent) sampling hypothesis by

4.3 $$P_{\theta }(W)=\prod_{i=1}^{I}\Phi \left(\sum_{\;j=1}^{J}w_{ij};\frac{J}{I},\theta \frac{J}{I}\left(1-\frac{1}{I}\right)\right),$$

where $\Phi \;(\cdot ;a,b^{2})$ is the p.d.f. of the normal law with mean *a* and variance *b*^2^, and *θ*( *J*/*I*)(1 − 1/*I*) is proportional to the multinomial variance up to the over-dispersion parameter *θ* > 0. The uniform sampling hypothesis amounts to assuming that explanatory sequences have equal chances to contribute to the set of response sequences. With *J* response sequences, there are *J* draws of an explanatory sequence (one for each response sequence) among *I* explanatory sequences. Thus, under the uniform sampling hypothesis, the total contribution $\sum_{\;j=1}^{J}w_{ij}$ of the explanatory sequence $S_{i}^{\left(0\right)}$ has expectation *J*/*I*.

The H1-*χ*^2^ shape measures the discrepancy between $\sum_{\;j=1}^{J}w_{ij}$ and its expected value *J*/*I* by

4.4 $$P_{\theta }(W)=\theta \chi^{2}\left(\sum_{i=1}^{I}\frac{{\left(\sum_{\;j=1}^{J}w_{ij}-J/I\right)}^{2}}{J/I};I-1\right),$$

where $\chi^{2}(\cdot ;I-1)$ is the p.d.f. of the *χ*^2^ law with *I* − 1 degrees of freedom, and *θ* > 0 measures the influence of the penalization.

The H2-normal shape can be used when estimates of the mean and standard deviation of the distance between any sequence collected from any recipient host and the closest sequence collected from its source, say $\;{\bar{d}}_{\mathrm{obs}}$ and $\sigma_{\mathrm{obs}}^{2}$, are available (these estimates can be obtained from contact tracing data). The H2-normal shape measures how likely it is that this mean distance for the host unit of interest is drawn from the normal distribution with mean $\;{\bar{d}}_{\mathrm{obs}}$ and variance $\sigma_{\mathrm{obs}}^{2}$:

4.5 $$P_{\theta }(W)=\theta \prod_{\;j=1}^{J}\Phi \left(\sum_{i=1}^{I}w_{ij}d(S_{\;j},S_{i}^{\left(0\right)});\;{\bar{d}}_{\mathrm{obs}},\sigma_{\mathrm{obs}}^{2}\right),$$

where *θ* > 0 measures the influence of the penalization.

Thereafter and whatever the penalization shape, *θ* is called the penalization parameter.

### (c) Estimation and calibration of parameters, and inference of transmissions

Consider *M* sets of sequences **S**_1_, …, **S**_*M*_ collected from *M* host units. In a first step, for each set of sequences **S**_*m*_ and each value of *θ* in a finite set *Θ* to be specified, the penalized pseudo-likelihoods *f*_*μ*,*θ*_(**S**_*m*_|**S**_*m*′_), for *m*′ ≠ *m*, are maximized with respect to *μ* (let ${\hat{\mu }}_{m^{\prime }}(\theta )$ denote the maximizer, i.e. the estimate, of *μ*). The most likely source for host unit *m* given *θ*, say $\hat{s}(m;\theta )$, is then the host unit *m*′ leading to the highest value of $f_{{\hat{\mu }}_{m^{\prime }}(\theta ),\theta }(S_{m}|S_{m^{\prime }})$:

$$\hat{s}(m;\theta )=\underset{m^{\prime }\neq m}{\mathrm{argmax}}\;f_{{\hat{\mu }}_{m^{\prime }}(\theta ),\theta }(S_{m}|S_{m^{\prime }}).$$

In a second step, the penalization parameter *θ* is calibrated by building and optimizing a criterion that compares contact information and inferred sources of infection $\hat{s}(m;\theta )$, for *m* in a set M⊂{1,…,M} of *training* hosts (this procedure can also be used in practice to select a penalization shape among a set of candidate functions as those proposed in equations (4.3)–(4.5)). Driven by the applications in this study, we introduce the two following criteria. First, consider the case where contact information consists of tracing contacts for hosts m∈M. We define the criterion to be maximized as the proportion of inferred transmissions that are consistent with contact tracing:

4.6 $$\;\tilde{\Theta }=\underset{\theta \in \Theta }{\mathrm{argmax}}\frac{1}{\left|M\right|}\sum_{m\in M}1(\hat{s}(m;\theta )\in C_{m}),$$

where |M| is the number of elements in M; **1**(*E*) = 1 if event *E* is true, zero otherwise; and $C_{m}$ is the set of hosts in {1, …, *M*} that have been in contact with *m*. Second, consider the case where contact information consists of the geographical distances between hosts in the training set M⊂{1,…,M}. We define the criterion to be minimized as the average distance between the training hosts and their inferred sources (if the sources are in the training set):

4.7 $$\;\tilde{\Theta }=\underset{\theta \in \Theta }{\mathrm{argmin}}\frac{\sum_{m\in M}\delta (m,\hat{s}(m;\theta ))1\{\hat{s}(m;\theta )\in M\}}{\sum_{m\in M}1\{\hat{s}(m;\theta )\in M\}},$$

where $\delta (m,\hat{s}(m;\theta ))$ is the geographical distance between host *m* and its suspected source $\hat{s}(m;\theta )$. Note that, in both cases, $\tilde{\Theta }$ may be a set of values (and not only a single value) if the criterion is optimal for several *θ* in *Θ*. This was the case in the applications that we tackled, since criteria in (4.6) and (4.7) have values in very limited discrete sets (e.g. {0, 1/5, 2/5, 3/5, 4/5, 1} in the Ebola application). Thus, in each application, $\tilde{\Theta }$ was obtained by computing the criterion on a regular grid of *θ* values and by retaining only values maximizing the criterion. We observed that small variations in *θ* did not impact the criterion value, as well as link intensities defined below in (4.8), and the mesh size of the grid was tuned accordingly. In further applications, the grid search could be improved in two directions: first, one could use an iterative numerical algorithm for the optimization; second, one could replace the maximum/minimum rule by a quantile rule (i.e. using a tolerance threshold).

In a third step, we assess the intensity of the link between *m* and *m*′ in {1, …, *M*} by the proportion of values of *θ* in $\tilde{\Theta }$ for which $\hat{s}(m;\theta )$ coincides with *m*′:

4.8 $$\frac{1}{\left|\;\tilde{\Theta }\right|}\sum_{\theta \in \;\tilde{\Theta }}1\{\hat{s}(m;\theta )=m^{\prime }\},$$

where $\left|\;\tilde{\Theta }\right|$ is the number of elements in $\;\tilde{\Theta }$. This intensity of the link between two host units is used to infer who infected whom or, from a more conservative perspective, who is the most related with whom. When several sequence fragments are available (like in the Ebola case study), the link intensity defined in equation (4.8) is computed for each fragment, and then averaged to obtain the overall link intensity. Future work could explore alternatives to the average (e.g. robust mean and median) for assessing link intensities from several fragments.

Model and inference specifications that were used for the three case studies are summarized in electronic supplementary material, table S9.

## Supplementary Material

Supplementary tables and figures

## Data accessibility

Data available at: http://doi.org/10.5281/zenodo.2543673 [37].
