# Supplementary material for: Inferring epidemiological links from deep sequencing data: a statistical learning approach for human, animal and plant diseases
Source: Philos Trans R Soc Lond B Biol Sci. 2019 May 6;374(1775):20180258. doi: 10.1098/rstb.2018.0258 (PMC6553606; doi:10.1098/rstb.2018.0258)
Supplement: Supplementary tables and figures [file rstb20180258supp1.pdf]

Electronic supplementary material

Inferring epidemiological links from deep sequencing data: a statistical learning approach for human, animal and plant diseases

Alamil M., Hughes J., Berthier K., Desbiez C., Thébaud G. and Soubeyrand S.

March 18, 2019

Table S1: Statistics about data corresponding to the three case studies, namely Influenza in pigs, Ebola in humans and a potyvirus in salsifies.

Statistics	Influenza		Ebola	Potyvirus
	Naive chain	Vaccinated chain		
Number of host units	10	13	58	27
Number of sequence fragments	1	1	31	1
Fragment length°	939	939	885†	438
Mean (SD) sequencing depth	41.3 (16.2)	58.3 (14.8)	14300 (17200)†	1550 (930)
Number of different variants	331	623	16.1†	278
Mean (SD) number of different variants per host unit	18.6 (7.0)	26.1 (9.4)	1.37 (0.64)†	10.3 (7.6)
Mean (SD) distance b/n variants*	3.31	3.61 (1.34)	2.42 (1.01)†	25.9 (6.6)
Mean (SD) within-host distance b/n variants*	1.17	2.80 (1.00)	1.37 (0.56)†	23.6 (3.4)

° Obtained after the removal of sites with missing values.
† Average over the 31 available sequence fragments.
* The (genetic) distance between (b/n) two sequence fragments is the number of different nucleotides.

Table S2: Contact information used for the reconstruction of transmission chains of Influenza in pigs and Ebola in humans. Note that host 401 was alone in group 3 of the vaccinated chain.

Outbreak	Contact information	Training host	Contact
Swine influenza	For 2 hosts in the last group	106	105, 108, 112
Naive chain		112	105, 108, 106
	For 2 hosts in groups 3 and 4	111	104, 116, 109
		108	109, 111, 105
Swine influenza	For 2 hosts in the last group	400	412, 414, 413
Vaccinated chain		413	412, 414, 400
	For 2 hosts in groups 3 and 4	401	409, 417
		416	401,415
Ebola	For 5 hosts among 58	G3817	G3729
		G3820	G3729
		G3821	G3729
		G3823	G3729
		G3851	G3752

Table S3: Mean difference between each sequence fragment of each training host and the closest sequence fragment in its source identified by contact tracing. The last two lines give, for each host, the average and the standard deviation of the mean difference over all sequence fragments. Figures lower than 0.0005 are denoted by 0 to facilitate the identification of significant positive values.

Fragment	G3817	G3820	G3821	G3823	G3851
500-1500	0	0	0.020	0	0.031
1000-2000	0	0.012	0	0	0
1500-2500	0	0	0	0	0
2000-3000	0	0.002	0	0	0
2500-3500	0.009	0	0.024	0.020	0.013
3000-4000	0	0	0	0	0
3500-4500	0.002	0	0.074	0	0.012
4000-5000	0	0	1.081	0	0
4500-5500	0	0	0.029	0	0.014
5000-6000	0	0.018	0	0	0
5500-6500	0	0	0	0	0
6000-7000	0	0	0.011	0	0
6500-7500	0	0	0	0	0
7000-8000	0	0.005	0	0	0.010
7500-8500	1.047	0.073	0.078	0	0
8000-9000	1.010	0	0	0	0.037
8500-9500	0	0	0	0	0
9000-10000	0	0	0.050	0	0
9500-10500	2.000	0	1.000	0	0
10000-11000	2.000	0	1.000	0	0
10500-11500	0	0	0.057	0	0.002
11000-12000	0	0.073	0	0.005	0.003
11500-12500	0	0	0	0	0
13000-14000	1.000	0	0	0	0
13500-14500	1.000	0	0	0	0
14000-15000	0	0.002	0	0	0
14500-15500	0	0	0	0	0
15000-16000	0	0	0	0	0
15500-16500	0	0	0.034	0	0
16000-17000	0	0	0	1.001	0.001
16500-17500	0	0	0.043	0.936	0
17000-18000	0	0	0	0	0
Average	0.252	0.006	0.109	0.061	0.004
SD	0.570	0.018	0.301	0.238	0.009

Table S4: Potential donors for training host G3817 whose donor identified with contact tracing is G3729. Link intensities and ranks were obtained by cross-validation.

Recipient	Donor	Link intensity	Rank
G3817	EM111	0.0409	1
	G3713	0.0398	2
	G3788	0.0393	3
	G3724	0.0391	4
	EM113	0.0273	5
	EM115	0.0266	6
	G3735	0.0257	7
	G3771	0.0257	7
	G3809	0.0257	7
	EM112	0.0250	10
	EM110	0.0248	11
	G3816	0.0248	12
	G3821	0.0248	12
	EM106	0.0247	14
	EM124	0.0246	15
	EM119	0.0242	16
	G3707	0.0238	17
	EM104	0.0237	18
	NM042	0.0237	18
	G3752	0.0237	20
	G3729	0.0237	21
	G3820	0.0232	22
	G3750	0.0225	23
	G3734	0.0218	24
	EM096	0.0215	25
	G3677	0.0214	26
	G3758	0.0213	27
	G3770	0.0212	28
	G3787	0.0208	29
	G3679	0.0206	30
	G3818	0.0203	31
	EM121	0.0202	32
	G3682	0.0200	33
	EM120	0.0189	34
	G3823	0.0185	35
	G3800	0.0180	36
	G3769	0.0178	37
	G3676	0.0173	38
	G3683	0.0169	39
	G3670	0.0162	40
	G3680	0.0151	41
	G3686	0.0142	42
	G3805	0.0074	43
	G3789	0.0033	44

Table S5: Potential donors for training host G3820 whose donor identified with contact tracing is G3729. Link intensities and ranks were obtained by cross-validation.

Recipient	Donor	Link intensity	Rank
G3820	G3676	0.0525	1
	G3729	0.0277	2
	G3677	0.0270	3
	EM121	0.0269	4
	EM113	0.0269	5
	G3734	0.0268	6
	G3707	0.0267	7
	EM096	0.0263	8
	G3679	0.0254	9
	G3788	0.0249	10
	G3724	0.0244	11
	EM115	0.0244	12
	G3682	0.0239	13
	G3735	0.0238	14
	G3771	0.0238	14
	G3809	0.0238	14
	G3758	0.0238	17
	G3823	0.0236	18
	EM120	0.0231	19
	G3787	0.0229	20
	EM110	0.0229	21
	G3750	0.0229	22
	G3816	0.0229	22
	G3821	0.0229	22
	EM106	0.0227	25
	G3713	0.0226	26
	EM104	0.0223	27
	G3769	0.0223	28
	EM112	0.0220	29
	NM042	0.0219	30
	G3800	0.0218	31
	EM124	0.0216	32
	EM119	0.0215	33
	EM111	0.0211	34
	G3752	0.0205	35
	G3683	0.0204	36
	G3818	0.0192	37
	G3770	0.0191	38
	G3680	0.0188	39
	G3817	0.0179	40
	G3686	0.0178	41
	G3670	0.0170	42
	G3805	0.0057	43
	G3789	0.0033	44

Table S6: Potential donors for training host G3821 whose donor identified with contact tracing is G3729. Link intensities and ranks were obtained by cross-validation.

Recipient	Donor	Link intensity	Rank
G3821	G3816	0.0556	1
	G3729	0.0513	2
	EM104	0.0391	3
	G3771	0.0344	4
	EM106	0.0338	5
	G3752	0.0305	6
	G3788	0.0277	7
	G3787	0.0269	8
	EM096	0.0259	9
	G3734	0.0258	10
	EM113	0.0248	11
	G3735	0.0248	11
	EM115	0.0245	13
	EM111	0.0239	14
	EM112	0.0235	15
	G3670	0.0234	16
	G3809	0.0234	17
	G3683	0.0232	18
	EM110	0.0230	19
	EM124	0.0222	20
	G3707	0.0214	21
	G3770	0.0213	22
	G3724	0.0211	23
	G3713	0.0211	24
	G3818	0.0210	25
	EM119	0.0209	26
	EM121	0.0203	27
	G3677	0.0200	28
	G3758	0.0196	29
	NM042	0.0194	30
	G3820	0.0188	31
	EM120	0.0188	32
	G3750	0.0187	33
	G3682	0.0180	34
	G3679	0.0177	35
	G3817	0.0174	36
	G3823	0.0173	37
	G3769	0.0166	38
	G3800	0.0162	39
	G3676	0.0147	40
	G3680	0.0124	41
	G3686	0.0113	42
	G3805	0.0068	43
	G3789	0.0016	44

Table S7: Potential donors for training host G3823 whose donor identified with contact tracing is G3729. Link intensities and ranks were obtained by cross-validation.

Recipient	Donor	Link intensity	Rank
G3823	G3682	0.0300	1
	EM106	0.0293	2
	G3769	0.0290	3
	EM104	0.0283	4
	G3677	0.0271	5
	EM113	0.0270	6
	G3820	0.0263	7
	EM121	0.0262	8
	G3683	0.0256	9
	G3735	0.0254	10
	G3771	0.0254	10
	EM096	0.0253	12
	G3707	0.0253	13
	G3729	0.0247	14
	EM115	0.0245	15
	G3724	0.0245	15
	G3758	0.0244	17
	G3788	0.0244	18
	G3679	0.0244	19
	G3821	0.0244	19
	G3734	0.0244	21
	EM120	0.0243	22
	G3787	0.0238	23
	G3809	0.0238	24
	EM112	0.0236	25
	EM110	0.0236	26
	EM124	0.0235	27
	G3750	0.0229	28
	G3816	0.0228	29
	G3800	0.0224	30
	G3713	0.0219	31
	EM111	0.0212	32
	NM042	0.0210	33
	G3818	0.0200	34
	G3676	0.0197	35
	G3752	0.0196	36
	EM119	0.0195	37
	G3770	0.0192	38
	G3817	0.0184	39
	G3680	0.0181	40
	G3670	0.0179	41
	G3686	0.0166	42
	G3805	0.0067	43
	G3789	0.0033	44

Table S8: Potential donors for training host G3851 whose donor identified with contact tracing is G3752. Link intensities and ranks were obtained by cross-validation.

Recipient	Donor	Link intensity	Rank	Donor	Link intensity	Rank
G3851	G3769	0.0438	1	G3800	0.0139	45
	G3825	0.0430	2	G3838	0.0135	46
	G3724	0.0326	3	G3670	0.0132	47
	EM106	0.0274	4	G3682	0.0131	48
	G3771	0.0264	5	EM120	0.0128	49
	G3829	0.0262	6	G3817	0.0127	50
	EM104	0.0255	7	G3683	0.0119	51
	G3821	0.0250	8	G3823	0.0118	52
	G3752	0.0244	9	G3676	0.0112	53
	G3850	0.0211	10	G3680	0.0105	54
	EM113	0.0208	11	G3686	0.0098	55
	G3848	0.0208	11	G3805	0.0057	56
	EM115	0.0206	13	G3789	0.0025	57
	G3826	0.0200	14			
	G3856	0.0198	15			
	EM111	0.0187	16			
	G3735	0.0177	17			
	G3809	0.0177	17			
	G3840	0.0177	17			
	G3788	0.0175	20			
	EM110	0.0170	21			
	G3816	0.0170	22			
	NM042	0.0169	23			
	EM112	0.0168	24			
	G3845	0.0168	25			
	G3677	0.0165	26			
	G3707	0.0163	27			
	EM124	0.0161	28			
	G3713	0.0161	29			
	G3729	0.0159	30			
	G3787	0.0158	31			
	G3820	0.0156	32			
	EM119	0.0155	33			
	G3841	0.0147	34			
	G3734	0.0146	35			
	G3770	0.0146	36			
	EM096	0.0145	37			
	G3679	0.0145	37			
	G3750	0.0145	39			
	G3758	0.0144	40			
	G3846	0.0144	41			
	G3831	0.0143	42			
	EM121	0.0142	43			
	G3818	0.0140	44			

Table S9: Specification of the components of the inference procedure. Note that setting Δ_{ij} at the value 1 implies that the substitution parameter μ corresponds, for each inferred transmission, to the expected number of substitutions per nucleotide in the evolutionary duration separating the two samples.

Model component	Influenza	Ebola	Potyvirus
Duration Δ_{ij}	$\Delta_{ij} \equiv 1$	$\Delta_{ij} \equiv 1$	$\Delta_{ij} \equiv 1$
Shape for P_θ	H1-Normal<br>(eq. (4.3))	H2-normal (eq. (4.5))<br>$(\bar{d}_{\mathrm{obs}}, \sigma^2_{\mathrm{obs}})$ estimated from training donor–recipient pairs	H1-Chi-squared<br>(eq. (4.4))
Set Θ of values for the penalisation parameter θ	Naive chain:<br>$\{0, 0.1, 0.2, \dots, 4\}$<br>Vaccinated chain:<br>$\{0, 0.25, 0.5, \dots, 10\}$	$\{0, 10, 20, \dots, 200\}$	$\{0, 1, 2, \dots, 40\}$
Basis for the calibration of θ	Contact tracing<br>(eq. (4.4))	Contact tracing<br>(eq. (4.6))	Geographical distance<br>(eq. (4.7))

Table S10: Discrepancy between inferred transmission graphs and reference graphs measured by the proportion of correct source identifications (CSI) and the Jeffreys discrepancy (JD) averaged over all hosts. For the Influenza case studies, the reference graph is the graph where the source for the hosts in the first group is an external source with probability 1, and the source for the hosts in the subsequent groups is any host in the preceding group with probability 0.5 (when the preceding group consists of 2 hosts) or probability 1 (when the preceding group consists of a single host; this occurs once in the vaccinated chain). For the Ebola case study, the reference is the graph obtained with BadTrIP by De Maio et al. (2018); in this case, the criteria were computed from recipient hosts that were in both analyses (BadTrIP and SLAFEEL). The proportion of CSI is computed as the proportion of hosts whose most likely source (based on the inferred graph) coincides with (one of) its source(s) in the reference graph (for the Ebola case study, the sources in the reference graph are only the most likely sources provided by BadTrIP; see Figure S14 for a less conservative definition). The JD (Chung et al., 1989; Jeffreys, 1946) measures the distance between two finite discrete probability distributions, say $\mathbf{p} = (p_1, \ldots, p_n)$ and $\mathbf{q} = (q_1, \ldots, q_n)$, by the quantity $\sum_{i=1}^{n}(\sqrt{p_i} - \sqrt{q_i})^2$. In our applications, $\mathbf{p}$ gives for a given recipient host the estimated probability for any other host to be its donor, and $\mathbf{q}$ gives for the same recipient host the reference vector of probabilities built as described above. For each inferred transmission graph, the JD was computed for all observed recipient hosts and, then, averaged.

Case study	Method	Penal.	Training hosts	Figure	Prop. CSI	Mean JD	SE JD
Swine Influenza	SLAFEEL	Yes	106, 112	1 (A)	0.60	0.84	0.19
Naive chain	SLAFEEL	Yes	111, 108	1 (B)	0.60	0.78	0.14
	SLAFEEL	No	–	S3 (A)	0.20	1.48	0.18
	BadTrIP*	–	–	S12 (A)	0.30	0.90	0.14
Swine Influenza	SLAFEEL	Yes	400, 413	1 (C)	0.42	0.86	0.19
Vaccinated chain	SLAFEEL	Yes	401, 416	1 (D)	0.42	0.90	0.24
	SLAFEEL	Yes	400, 413, 401	S1 (A)	0.50	1.02	0.25
	SLAFEEL	Yes	400, 413, 415	S1 (B)	0.50	1.01	0.26
	SLAFEEL	Yes	400, 413, 416	S1 (C)	0.42	1.11	0.27
	SLAFEEL	Yes	401, 415, 416	S1 (D)	0.42	0.90	0.24
	SLAFEEL	No	–	S3 (B)	0.42	0.92	0.22
	BadTrIP*	–	–	S12 (B)	0.33	0.99	0.22
Ebola	SLAFEEL *vs* BadTrIP	Yes	G3817, G3829 G3821, G3823 G3851	3	0.08	0.80	0.04

*To fairly compare BadTrIP and SLAFEEL in the Influenza case studies, we *a posteriori* pruned impossible transmissions inferred by BadTrIP based on temporal information (as we *a priori* did with SLAFEEL), we reweighted the remaining inferred transmissions such that their probabilities sum to 1 for each infected host, and we computed the CSI, the mean JD and the SD of JD from the remaining transmissions and their updated probabilities.

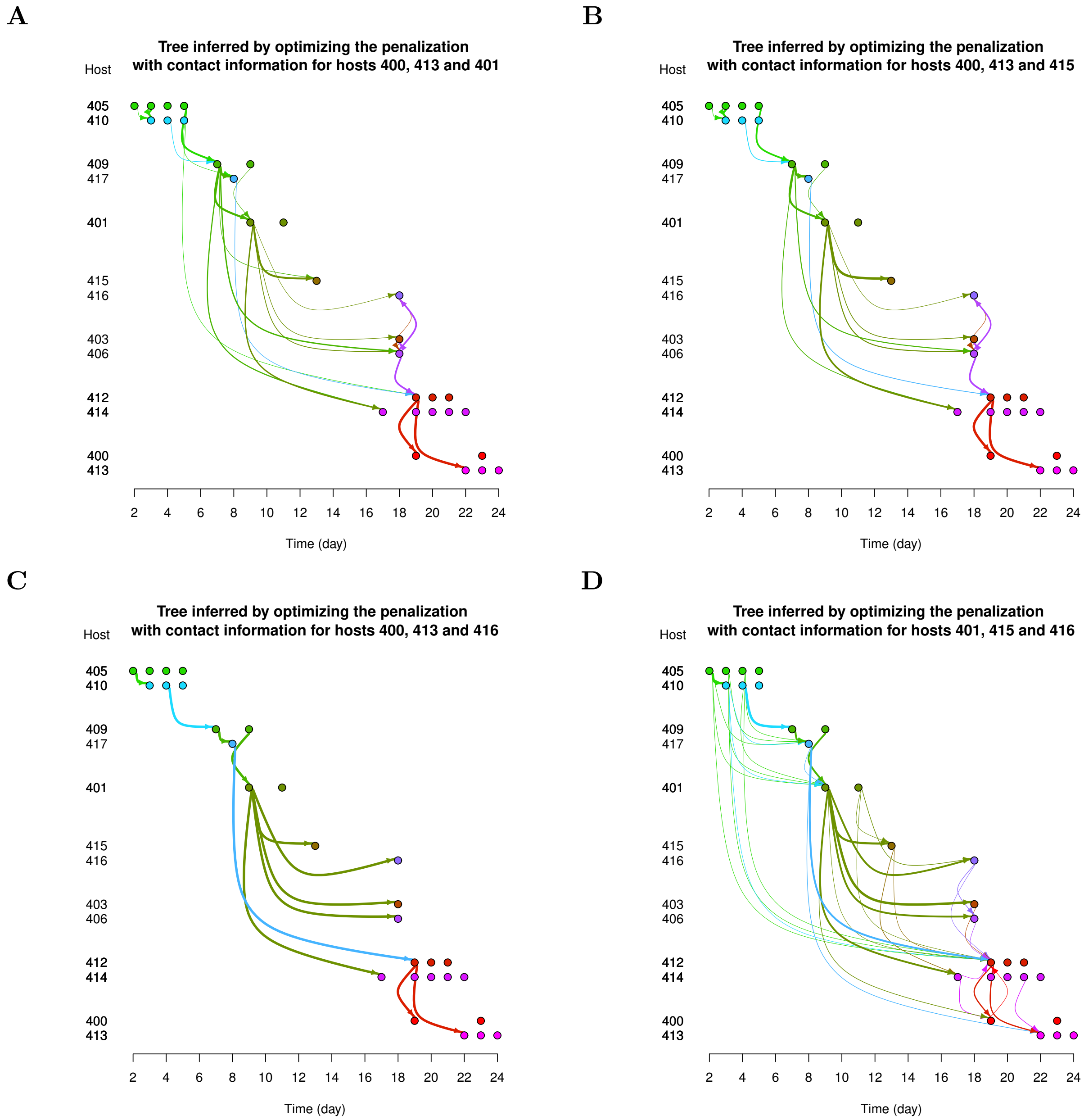

Figure S1: Transmissions inferred in the vaccinated chain with different sets of three training hosts for calibrating the penalisation. The thickness of each arrow is proportional to the intensity of the corresponding link.

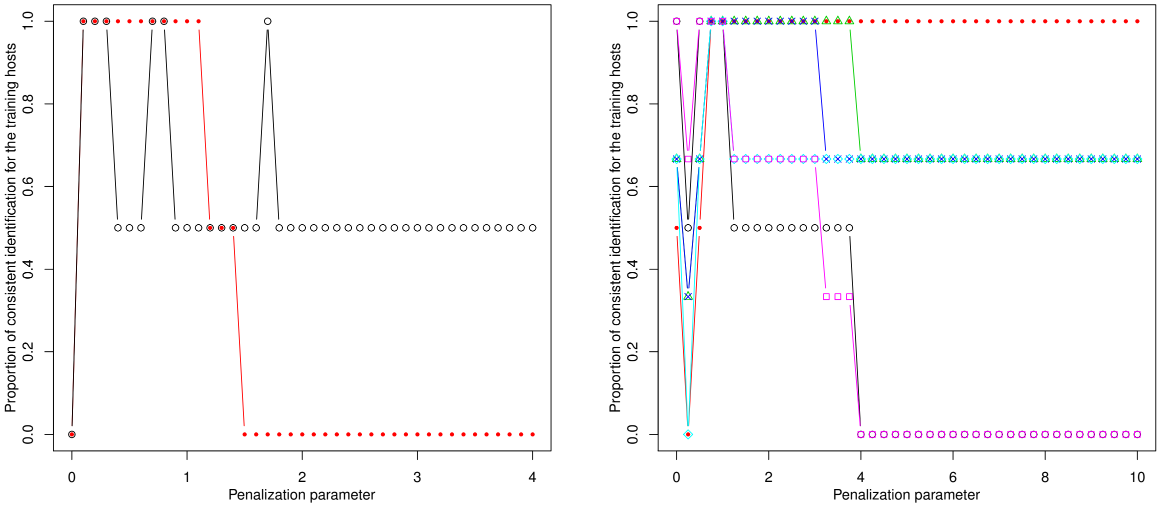

Figure S2: Proportion of source identifications that are consistent with contact information about the training hosts for the naive chain (left) and the vaccinated chain (right), as a function of the penalisation parameter. In each panel, the rate of consistent identifications is shown in red when the training hosts are the two pigs of the last group of the outbreak, and in black when the training hosts are two pigs selected from the 3rd and 4th groups of the outbreak; see details in Table 1 of the main text. In the right panel, the green curve corresponds to training hosts 400, 413 and 401; the dark blue curve to 400, 413 and 415, the light blue curve to 400, 413 and 416 and the pink curve to 401, 415 and 416. Adding a third host to training data allows us to reduce the range of optimal penalisation parameters.

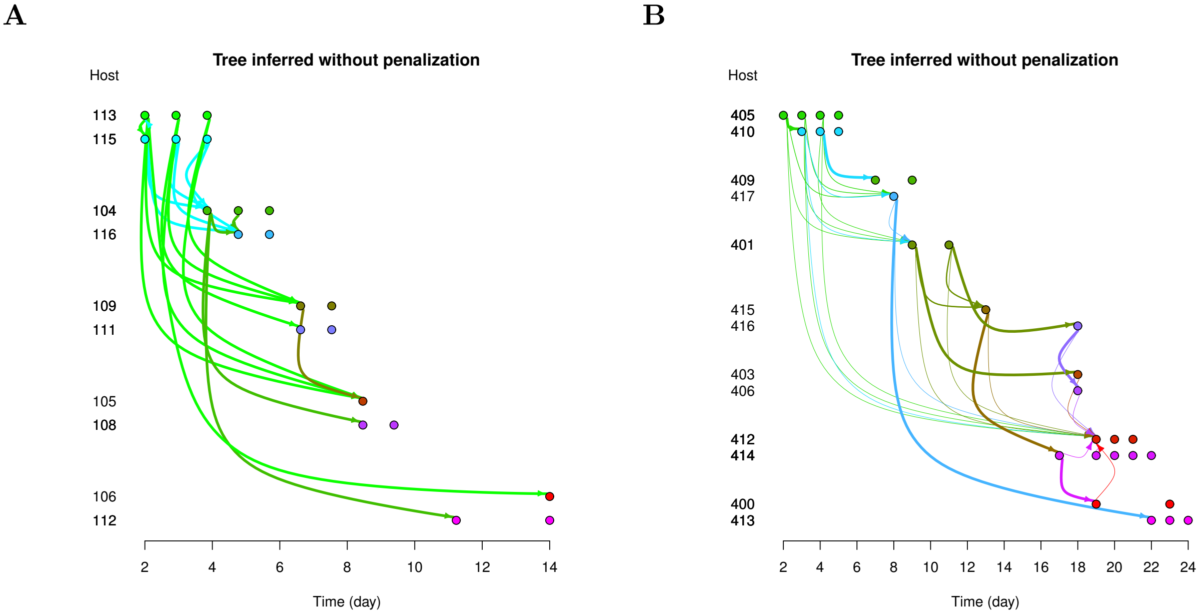

Figure S3: Transmissions inferred in the naive chain (left) and vaccinated chain (right) without including the penalisation and, therefore, without including training hosts.

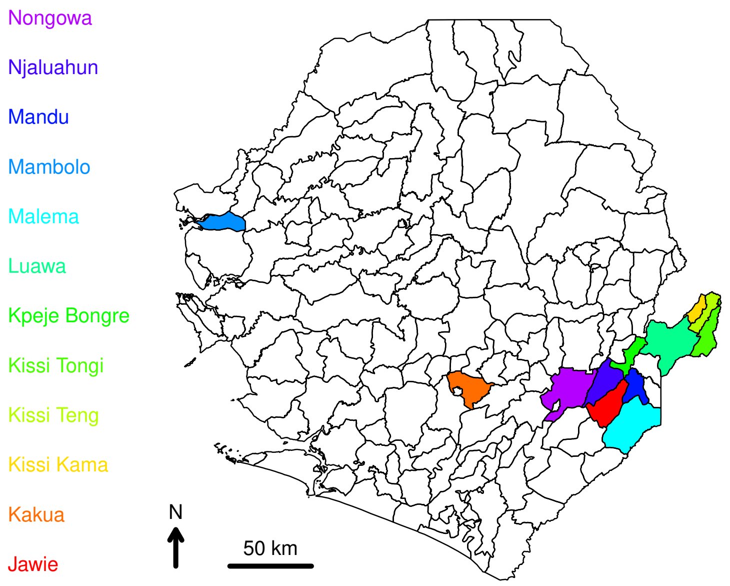

Figure S4: Map of Sierra Leone showing the locations of chiefdoms included in the analysis of Ebola data.

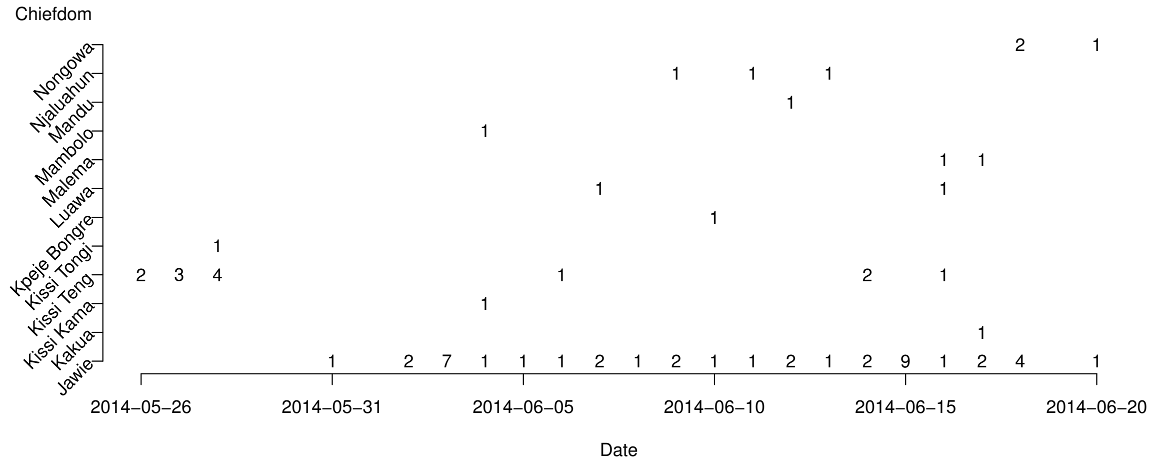

Figure S5: Number of Ebola patients included in the analysis as a function of collection date and chiefdom.

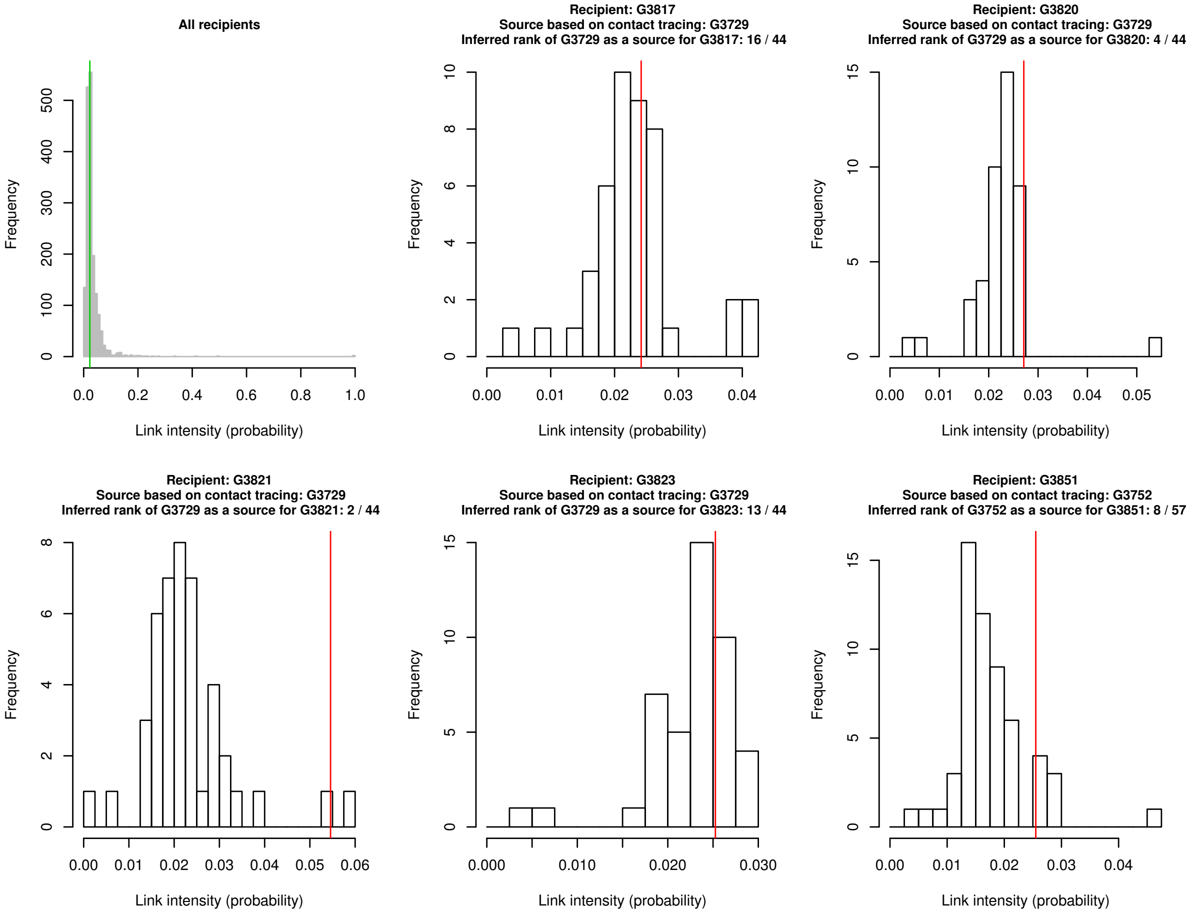

Figure S6: Estimated intensities of links in the Ebola dataset for all recipients (top left panel; green line: median intensity) and for each recipient in the training set of hosts (other panels; red line: intensity for the source identified with contact tracing). This figure was obtained from the combined analysis of 31 sequence fragments and without cross-validation.

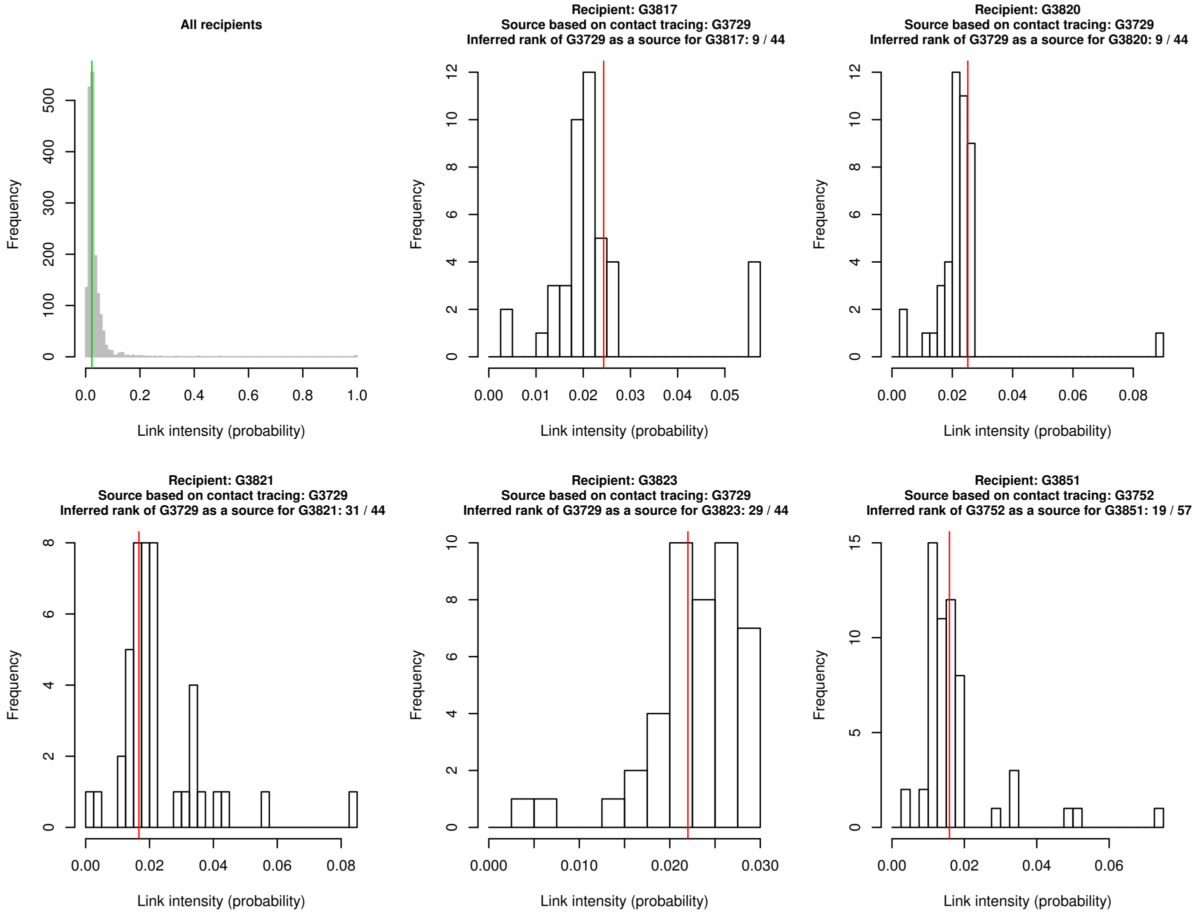

Figure S7: Estimated intensities of links for all recipients (top left panel; green line: median intensity) and for each recipient in the training set of hosts (other panels; red line: intensity for the source identified with contact tracing). This figure was obtained from the combined analysis of 15 sequence fragments from sequence site 500 to sequence site 9000, and with cross-validation.

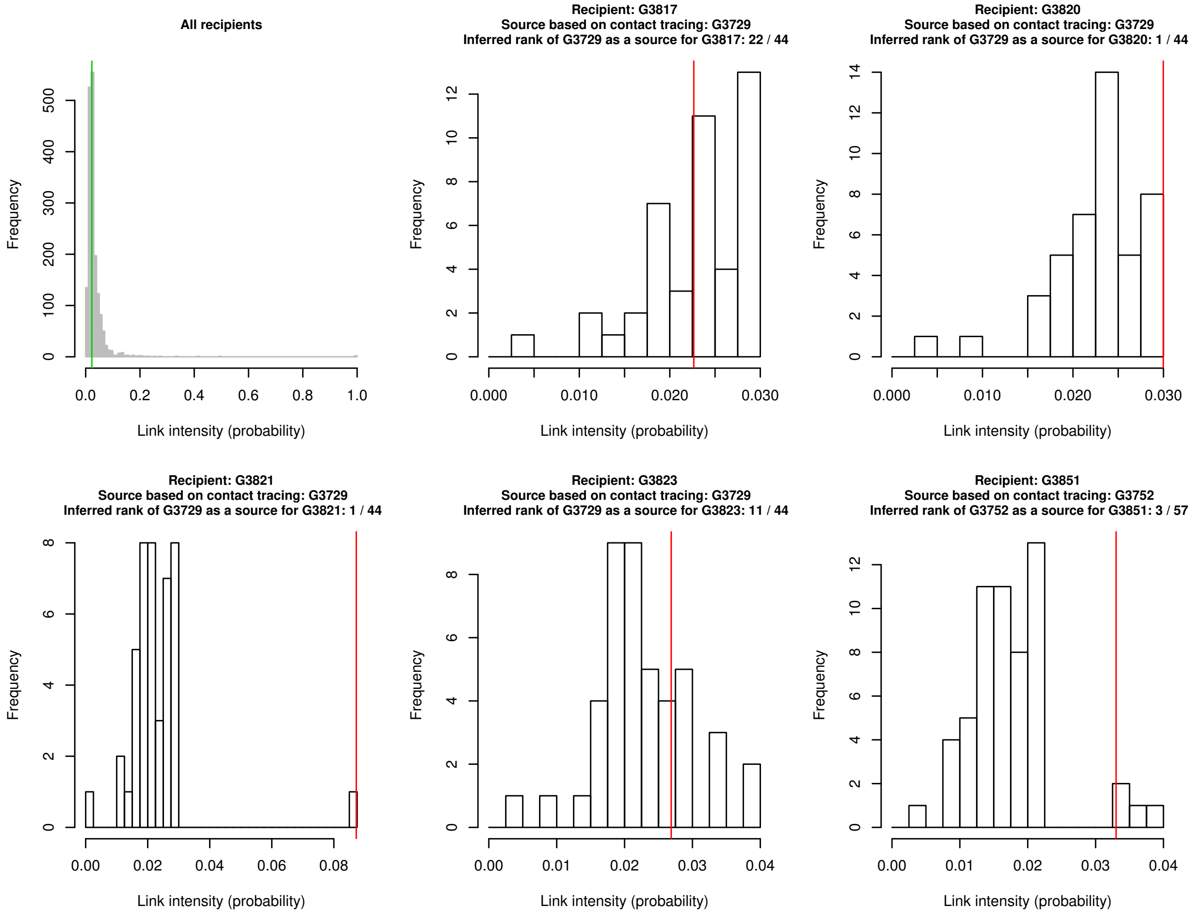

Figure S8: Estimated intensities of links for all recipients (top left panel; green line: median intensity) and for each recipient in the training set of hosts (other panels; red line: intensity for the source identified with contact tracing). This figure was obtained from the combined analysis of 15 sequence fragments from sequence site 9000 to sequence site 18000, and with cross-validation.

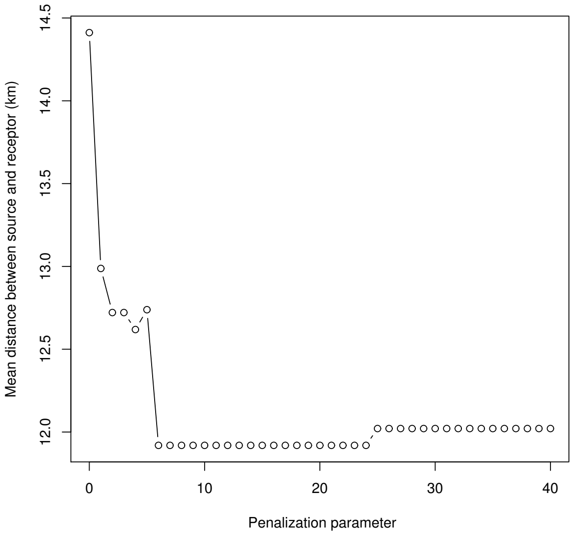

Figure S9: Mean distance between connected salsify patches with respect to the penalisation parameter.

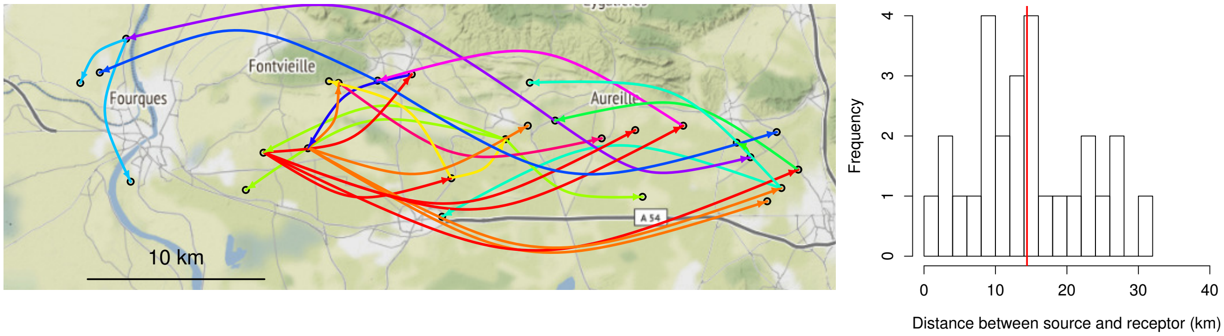

Figure S10: Links inferred without penalisation between salsify populations based on sampled sets of potyvirus sequences (left; links from the same source have the same color) and distribution of link distances (right; the vertical red line indicates the mean distance).

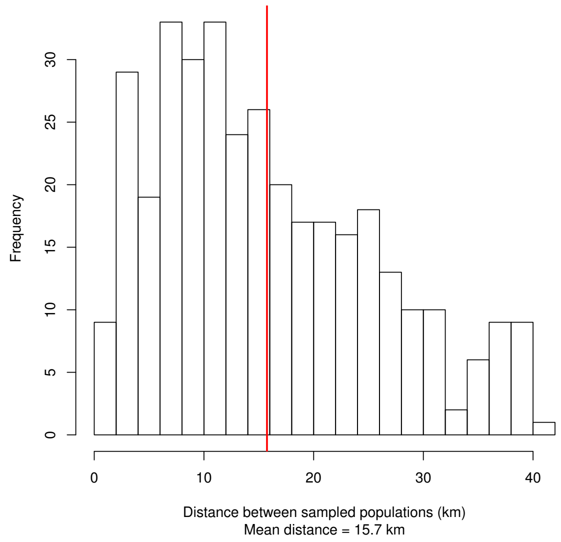

Figure S11: Distribution of distances between salsify patches. The vertical red line indicates the mean distance.

A

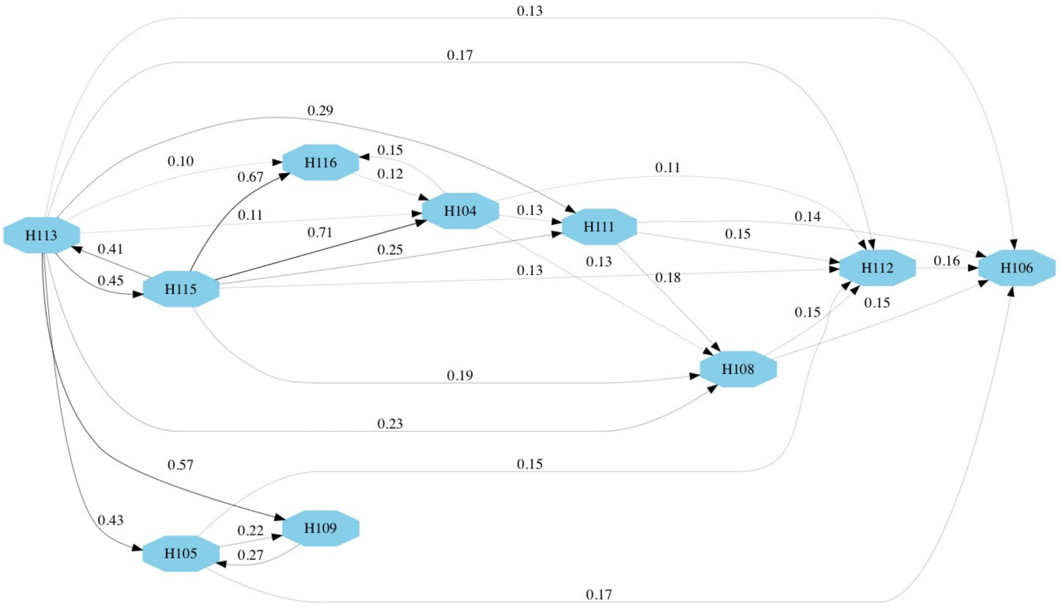

B

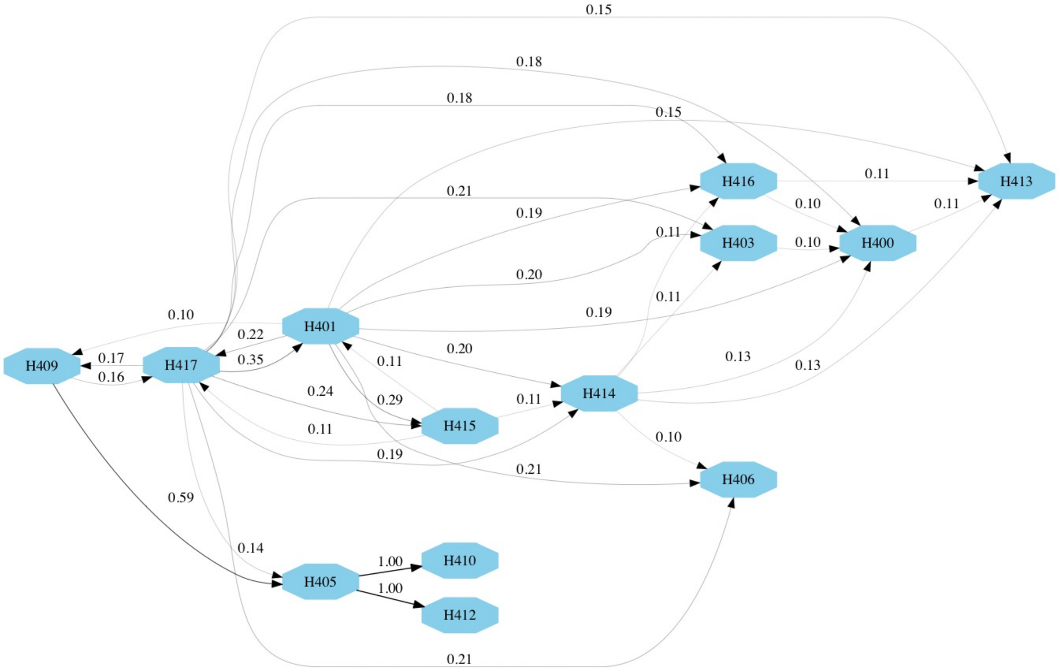

Figure S12: Inference of transmissions in the naive (A) and vaccinated (B) Swine influenza transmission chains. Transmission events with posterior probability higher than 0.10 as inferred by BadTrIP are shown. Hexagons represent hosts, while arrows are transmission events between hosts. The posterior probability of transmissions are shown next to the arrows and higher values are shown with thicker arrows. — For both datasets, the sequences for each sample were re-coded for use in the BadTrIP package (De Maio et al., 2018) embedded in BEAST2 (Bouckaert et al., 2014). BadTrIP uses the PoMo model (De Maio et al., 2015) that describes how a population evolves along the branches of a population tree. We allowed each host in the Swine influenza transmission chain to be infectious for the whole period of the experiment. We ran the BadTrIP MCMC for approximately 4 million independent steps, which provided an effective sample size of 20 and took one week of computation (on one CPU of an iMac 4 GHZ Intel Core i7).

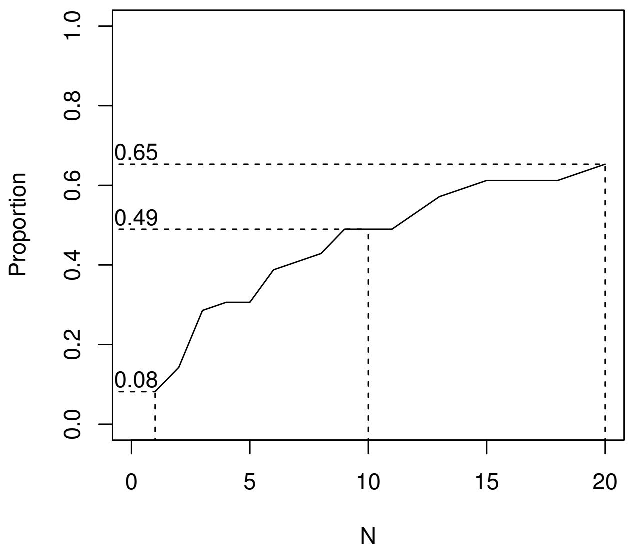

Figure S13: Proportion of recipient hosts whose SLAFEEL-based most likely sources are among the N BadTrIP-based most likely sources. The proportion obtained when N=1 corresponds to the proportion of correct source identifications (CSI) provided in Table S10.

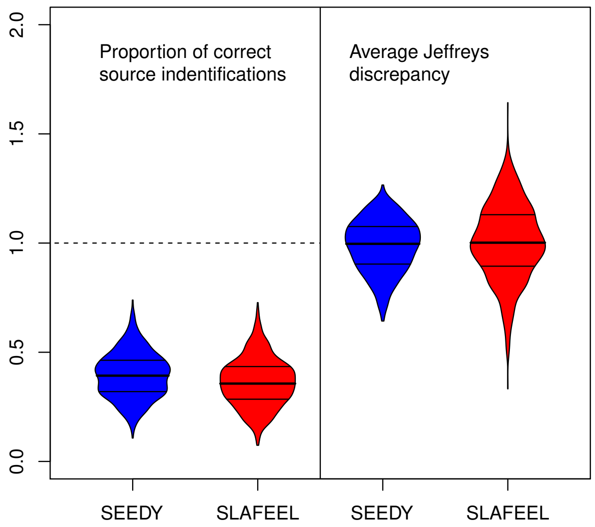

Figure S14: Comparison between SLAFEEL and SEEDY (Worby and Read, 2015) in their ability to identify transmission trees simulated with SEEDY. The comparison was made by assessing the discrepancy between inferred transmission graphs and the simulated graphs, using two criteria: the proportion of correct source identifications and the average Jeffreys discrepancy (see their definitions in table S10). These criteria were computed for 1000 data sets generated with SEEDY by using parameter values chosen by Worby and Read to generate their 4th figure (see details below). The mean epidemic size of simulated outbreaks was: 26.6 infected hosts (SD=2.3). For the application of SLAFEEL to each simulated outbreak, we randomly drew 4 training hosts whose sources were supposed to be known, we chose the H1-normal penalisation, we set $\Delta_{ij} \equiv 1$ and $\Theta = \{0, 1, 2, \ldots, 10\}$. — Outbreaks were simulated with the following parameter values. Number of susceptibles in population: 30. Rate of infection: 0.02. Rate of removal/recovery: 0.001. Mutation rate per sequence per generation: 0.001. Equilibrium population size within host: 1000. Transmission bottleneck size: 10. Samples taken per time point: 10 (1 time point per host, randomly and uniformly drawn between 1 and 300 time steps after host infection). Minimum number of cases before returning (retries until fulfilled): 20. Genome length: 10^5.

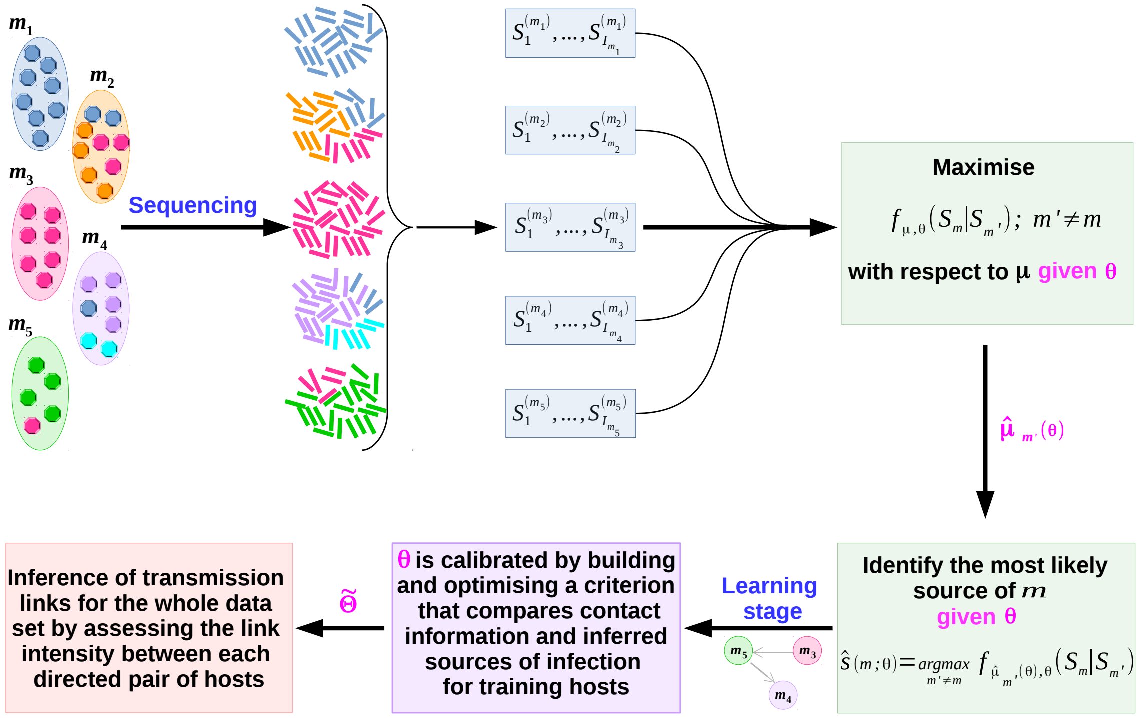

Figure S15: Graphical representation of SLAFEEL. Virus sequences are collected from several hosts $m_1, m_2, \ldots$. In a first step, the penalised pseudo-likelihood $f_{\mu,\theta}(\mathbf{S}_m \mid \mathbf{S}'_m)$ is maximised for each possible donor–recipient pair (m', m) and a set of values for the penalisation parameter θ. This maximisation provides an estimate $\hat{\mu}_{m'}(\theta)$ of the evolutionary parameter μ given θ and the putative source m'. Then, given θ, the most likely source of the recipient m, say $s(m;\theta)$, is identified by maximising $f_{\hat{\mu}_{m'}(\theta),\theta}(\mathbf{S}_m \mid \mathbf{S}'_m)$ with respect to m'. In a second step, by using contact information about training hosts (e.g., m_3 possibly infected m_5 and m_5 possibly infected m_4), the penalization parameter θ is calibrated with a learning approach by building and optimising a criterion that compares contact information and sources of infection $\hat{s}(m_4;\theta)$ and $\hat{s}(m_5;\theta)$ inferred for training hosts m_4 and m_5, respectively. $\tilde{\Theta}$ is the set of penalisation values for which the criterion is optimal. In a third step, the link intensity is used to assess the likelihood of the link between a donor and a recipient.

References

Bouckaert, R., J. Heled, D. Kühnert, T. Vaughan, C.-H. Wu, D. Xie, M. A. Suchard, A. Rambaut, and A. J. Drummond (2014). BEAST 2: A software platform for Bayesian evolutionary analysis. *PLoS Computational Biology 10*, e1003537.

Chung, J. K., P. L. Kannappan, C. T. Ng, and P. K. Sahoo (1989). Measures of distance between probability distributions. *Journal of Mathematical Analysis and Applications 138*, 280–292.

De Maio, N., D. Schrempf, and C. Kosiol (2015). PoMo: An allele frequency-based approach for species tree estimation. *Systematic Biology 64*, 1018–1031.

De Maio, N., C. J. Worby, D. J. Wilson, and N. Stoesser (2018). Bayesian reconstruction of transmission within outbreaks using genomic variants. *PLoS Computational Biology 14*, e1006117.

Jeffreys, H. (1946). An invariant form for the prior probability in estimation problems. *Proceedings of the Royal Society of London A 186*, 453–461.

Worby, C. J. and T. D. Read (2015). 'SEEDY'(simulation of evolutionary and epidemiological dynamics): An R package to follow accumulation of within-host mutation in pathogens. *PLoS One 10*(6), e0129745.
